# Demographic characteristics, diagnostic challenges, treatment patterns, and caregiver burden of mitochondrial diseases: a retrospective cross-sectional study

**DOI:** 10.1186/s13023-024-03289-5

**Published:** 2024-08-02

**Authors:** Xutong Zhao, Meng Yu, Wei Zhang, Yue Hou, Yun Yuan, Zhaoxia Wang

**Affiliations:** 1 Department of Neurology, Beijing Jishuitan Hospital, Capital Medical University, Beijing, 102208 China; 2 Department of Neurology, Peking University First Hospital, Beijing, 100034 China; 3 Department of Geriatrics, Peking University First Hospital, Beijing, 100034 China

**Keywords:** Mitochondrial disease, Disease burden, Demographics, Diagnostic challenges, Treatment patterns, Caregiver burden

## Abstract

**Background:**

This study aimed to explore the demographic characteristics, diagnostic challenges, treatment patterns, and caregiver burden of mitochondrial diseases.

**Methods:**

This retrospective cross-sectional study enrolled patients diagnosed with mitochondrial diseases from the Department of Neurology at Peking University First Hospital between January 2010 and December 2021. A questionnaire covering demographic characteristics, diagnostic dilemma, treatment, economic aspects, and caregiver stress was administered, and disability was assessed using the modified Rankin Scale (mRS).

**Results:**

A total of 183 patients (mean age: 16 (IQR: 12–25), 49.72% males) were enrolled, including 124 pediatric patients and 59 adult patients. MELAS (106. 57.92%) and Leigh syndrome (37, 20.22%) were predominant among the mitochondrial disease subtypes. Among them, 132 (72.13%) patients were initially misdiagnosed with other diseases, 58 (31.69%) patients visited 2 hospitals before confirmed as mitochondrial disease, and 39 (21.31%) patients visited 3 hospitals before confirmed as mitochondrial disease. Metabolic modifiers were the most common type of drugs used, including several dietary supplements such as L-carnitine (117, 63.93%), Coenzyme Q10 (102, 55.74%), idebenone (82, 44.81%), and vitamins (99, 54.10%) for proper mitochondrial function. Mothers are the primary caregivers for both children (36.29%) and adults (38.98%). The mRS score ranged from 0 to 5, 92.35% of the patients had different degrees of disability due to mitochondrial disease. The average monthly treatment cost was 3000 RMB for children and 3100 RMB for adults.

**Conclusions:**

This study provided valuable insights into the characteristics and challenges of mitochondrial diseases, which underscores the need for improved awareness, diagnostic efficiency, and comprehensive support for patients and caregivers.

## Background

Mitochondrial disease, characterized by defective oxidative phosphorylation, presents diverse clinical phenotypes [1–3]. Given that mitochondria are ubiquitous except in mature erythrocytes, this genetic disorder affects various systems. Mitochondrial disease often affects organs with high energy demands, such as the brain, skeletal muscles, and heart [4–6]. Globally, mitochondrial disease affects around 1 in 5000 adults [4, 7], yet epidemiological data for China are currently lacking. Common mitochondrial disease forms include mitochondrial myopathy [8], encephalopathy [9], lactic acidosis [5], stroke-like episodes (MELAS) [5], Kearns-Sayre syndrome (KSS syndrome) [10, 11], chronic progressive extraocular muscle paralysis (CPEO) [12, 13], and Leigh syndrome [14, 15].

Diagnosis is challenging due to varied symptoms and signs. A comprehensive evaluation, considering clinical symptoms, imaging reports, biochemical indexes, muscle biopsy results, and genetic mutations, is essential. Lack of sensitive biomarkers, coupled with the cost and invasiveness of certain tests, complicates the diagnostic process [16]. The progressive clinical course often leads to poor functional and survival outcomes, imposing a substantial burden on patients and caregivers [16].

Supportive therapies involve metabolic coenzymes, antioxidants, and energy substitutes. Dietary modifications, nutritional supplements, and exercise therapy are also employed, with varying effectiveness among patients [17]. Despite these interventions, there is still no FDA-approved treatment for mitochondrial disease. Consequently, the disease burden is extensive, impacting psychological well-being and imposing significant direct and indirect healthcare costs on patients and communities [18]. Several studies have highlighted the adverse effects of mitochondrial disease on patients’ and families’ quality of life [19], the considerable psychological burden on caregivers of pediatric patients [20], and the associated financial expenses [7]. However, there is a lack of reports on the specific burden faced by patients and families in China.

Therefore, this study aimed to investigate the demographic characteristics, diagnostic challenges, treatment patterns, and caregiver burden of mitochondrial diseases.

## Methods

### Study design and patients

This retrospective cross-sectional study enrolled patients diagnosed with mitochondrial diseases from the Department of Neurology at Peking University First Hospital between January 2010 and December 2021. Inclusion criteria: (1) adult or pediatric patients with confirmed mitochondrial disease through gene detection and/or muscular tissue biopsy; (2) adult patients or pediatric patients’ caregivers who voluntarily participated and provided signed informed consent. Exclusion criteria encompassed patients or caregivers unable to complete the questionnaire. The study was approved by the ethics committee of the Peking University First Hospital. Informed consents were obtained from all adult participants and caregivers of pediatric patients.

### Data collection and definitions

All eligible adult patients and caregivers of eligible pediatric patients were provided an online questionnaire, developed by the Department of Neurology at Peking University First Hospital. The questionnaire, based on a prior study [21], comprised 50 questions and took approximately 30 min to complete. In the first section, the questionnaire covered fundamental demographic characteristics, including the patient’s age, gender, address, ethnicity, initial symptom, genotype and phenotype, age of onset, and educational background of the patient and family members. The second section included inquiries about the misdiagnosis of the disease, the number of hospitals visited before the diagnosis, current medication, and difficulties encountered during medical treatment. The third part featured questions related to the cost of illness, treatment effectiveness, and the disease burden on patients and their families. The disease burden primarily encompassed diagnostic difficulties (delays in diagnosis), financial costs, and caregiver stress.

The disability of patients was evaluated via in-person visit or telephone conversation using the modified Rankin Scale (mRS), in which 0 point means “completely asymptomatic”, 1 point means “no significant dysfunction and able to perform all daily duties and activities, despite symptoms”, 2 points means “mild disability, unable to complete all regular activities, but does not need assistance and can take care of their own needs”, 3 points means “moderate disability, requires some assistance, but does not need help for walking”, 4 points means “severe disability, unable to walk independently, unable to meet their own needs without help from others”, and 5 points means “severe disability, bedridden, incontinence, requiring constant care and attention”, 6 points means “death”.

### Statistical analysis

The statistical analysis was performed using GraphPad Prime 9 (USA). Continuous data with a normal distribution were described as mean ± standard deviation (SD); otherwise, they were presented as medians (interquartile range, IQR). Categorical data were described as n (%).

## Results

### Demographic characteristics

**Fig. 1:**
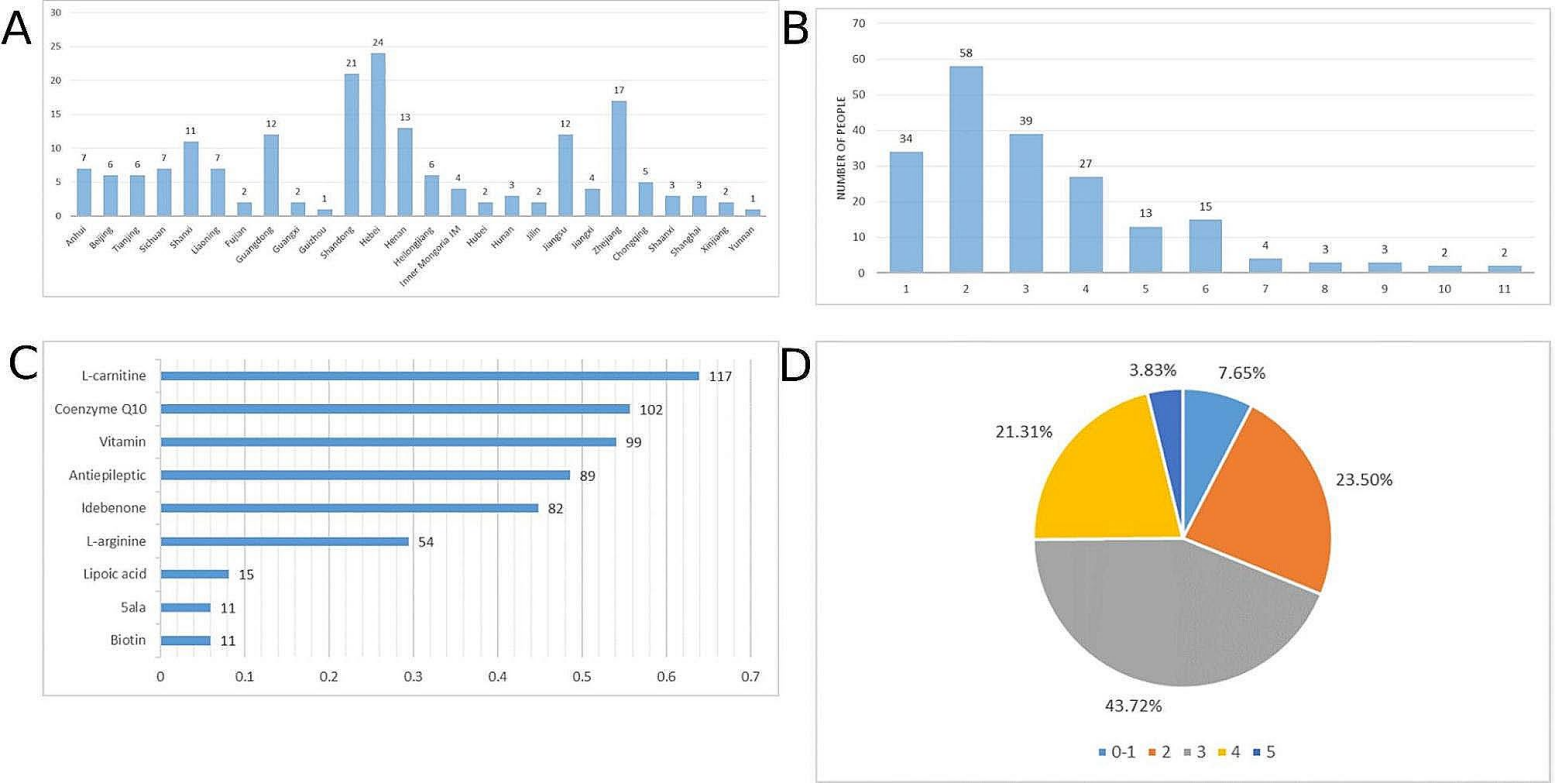
Disease burden of patients with mitochondrial disease. **(A)** The geographical distribution of patients with mitochondrial disease. **(B)** The number of hospitals visited by patients before confirmative diagnosis of mitochondrial disease. **(C)** Current medication status of patients. **(D)** The mRS score of patients in this study

A total of 222 questionnaires were collected, among them, 39 questionnaires were excluded due to incomplete answers, and finally a total of 183 patients (mean age: 16 (IQR: 12–25), 49.72% males) were enrolled into subsequent analysis, including 124 pediatric patients and 59 adult patients. Pediatric patients had a median age of 9 years old (IQR: 5–12), while adults had a median age of 26 years old (IQR: 21–38). Male patients constituted 51.61% of the pediatric cohort and 44.06% of the adult cohort. 95.97% of pediatric patients and 89.83% of adults were identified as Han ethnicity. Pediatric patients exhibited an earlier age of onset (median: 5 years, IQR: 1–8) compared to adults (median: 16 years, IQR: 12–27). Familial mitochondrial disease history was present in 20.16% of pediatric patients and 16.95% of adults. 94.35% of pediatric patients had an illiterate or primary school education, while 50.85% of adults completed high school or college. A majority of pediatric patients’ parents (66.13%) had high school or college education, compared to 37.29% of adult patients’ parents. Most pediatric patients (74.19%) and 38.98% of adults received confirmation of mitochondrial disease within 1–5 years. Pediatric patients had an average family monthly income of 3500 RMB (IQR: 2500–5000), whereas adults had a median of 3000 RMB (IQR: 2000–5000) **(**Table 1**)**. The patients came from 27 provinces in China, with Hebei having the highest number (24 patients, 13.11%), followed by Shandong (21 patients, 11.48%), Zhejiang (17 patients, 9.29%), and Guangdong (12 patients, 6.56%) **(**Fig. 1A**)**.

**Table 1:** Basic demographic characteristics

Characteristics	Children (n=124)	Adults (n=59)
Age (years), Median (IQR)	9 (5–12)	26 (21–38)
Gender, male, n (%)	64 (51.61%)	26 (44.06%)
Ethnic, Han	119 (95.97)	53 (89.83)
Age of onset (years), Median (IQR)	5 (1–8)	16 (12–27)
Family history of MD	25 (20.16)	10 (16.95)
Education level		
Illiterate/primary school	117 (94.35)	9 (15.25)
Middle school	7 (5.65)	20 (33.90)
High school/college	0	30 (50.85)
Parents’ education level		
Literate/primary school	5 (4.03)	11 (18.64)
Middle school	37 (29.84)	26 (44.07)
High school/college	82 (66.13)	22 (37.29)
Time since disease confirmation		
< 1 years, n (%)	2 (1.61)	1 (1.69)
1–5 years, n (%)	92 (74.19)	23 (38.98)
5–10 years, n (%)	28 (22.58)	21 (35.59)
> 10 years, n (%)	2 (1.61)	14 (23.73)
Average monthly income (RMB)	3500 (2500, 5000)	3000 (2000, 5000)

### Subtypes of mitochondrial disease

Among the 183 patients, there were 106 cases (57.92%) of MELAS, 37 cases (20.22%) of Leigh, 7 cases (3.83%) of MERRF, 2 cases (1.09%) of CPEO, 2 cases (1.09%) of KSS, 1 case (0.55%) of MELAS overlap Leigh, 1 case (0.55%) of LTBL, 3 cases (1.64%) of mitochondrial myopathy, 3 cases (1.64%) of mitochondrial DNA depletion syndrome, 2 cases (1.09%) of mitochondrial white matter encephalopathy, 6 cases (3.29%) of mitochondrial complex deficiency, 2 cases (1.09%) of nuclear gene-related mitochondrial disease, 10 cases (5.46%) of mitochondrial synthase deficiency, 2 patients (1.09%) had Nuclear Gene-Related Mitochondrial Disease, 1 case (0.55%) patients was Complex Oxidative Phosphorylation Defect Type **(**Table 2**)**.

**Table 2:** Mitochondrial disease subtypes

Disease subtypes	Cases (*n*)	Percentage (%)
MELAS	106	57.92
Leigh	37	20.22
MERRF	7	3.83
CPEO	2	1.09
KSS	2	1.09
MELAS overlap Leigh	1	0.55
LTBL	1	0.55
Mitochondrial myopathy	3	1.64
Mitochondrial DNA depletion syndrome	3	1.64
Mitochondrial Complex I deficiency	4	2.19
Mitochondrial Complex II deficiency	1	0.55
Mitochondrial Complex III deficiency	1	0.55
Mitochondrial white matter encephalopathy	2	1.09
Mitochondrial synthase deficiency	10	5.46
Nuclear gene-related mitochondrial disease	2	1.09
Complex oxidative phosphorylation defect type	1	0.55

### Mitochondrial disease diagnostic delay

A total of 132 (72.13%) patients were initially misdiagnosed with other diseases, such as viral encephalitis, epilepsy, dwarfism, myasthenia gravis, methylmalonic acidemia, cerebral infarction, cerebral palsy, strabismus, metachromatic and leukodystrophy. The number of hospitals visited by patients before confirmative diagnosis varied from 1 to 11, 58 (31.69%) patients visited 2 hospitals before confirmed as mitochondrial disease, 39 (21.31%) patients visited 3 hospitals before confirmed as mitochondrial disease, 34 (18.58%) patients visited 1 hospital before confirmed as mitochondrial disease **(**Fig. 1B**)**. The median interval between the first symptom and confirmative diagnosis was 1.44 (range: 0–17) years.

### Drug costs of mitochondrial disease

In this study, metabolic modifiers were the most common type of drugs used, including several dietary supplements such as L-carnitine (117, 63.93%), Coenzyme Q10 (102, 55.74%), idebenone (82, 44.81%), and vitamins (99, 54.10%) for proper mitochondrial function. Antiepileptics (89, 48.63%) were the second most prescribed drugs for mitochondrial disease patients, including levetiracetam, topiramate, clonazepam, oxcarbazepine, lamotrigine, valproate, and phenobarbital. In addition, 54 (29.51%) of patients took L-arginine. A few patients were given calcium, lipoic acid (15, 8.20%), and traditional Chinese medicine (Fig. 1C**)**.

### Caregivers’ burden

Mothers are the primary caregivers for both children (36.29%) and adults (38.98%), followed by fathers, parental co-care, grandparents, and other family members. The majority of families rely on unstable jobs for income, with 63.71% of children and 64.41% of adults. Fathers are predominantly responsible for the family’s economy, with 56.45% of children and 33.89% of adults. A small percentage of adults (3.39%) receive social assistance. Pre-diagnosis costs are relatively consistent, with median values of 7000 (3000, 15,000) for children and 6000 (4000, 10,000) RMB for adults **(**Table 3**)**. 77.05% of the patients had been hospitalized once or more for mitochondrial disease. The cost of hospitalization for mitochondrial disease is 30,000 RMB (IQR: 10,000–50,000) per year. The mRS score ranged from 0 to 5 in this study, and 92.35% of the patients had different degrees of disability due to mitochondrial disease (Fig. 1D).

**Table 3:** Caregivers’ burden

	Children (*n* = 124)	Adults (*n* = 59)
Take care of patients, n (%)		
Mother	45 (36.29)	23 (38.98)
Father	20 (16.13)	15 (25.42)
Parental co-care	22 (17.74)	7 (11.83)
Grandparents	33 (26.61)	7 (11.83)
Other members	4 (3.23)	7 (11.83)
Major sources of income		
Stable job	45 (36.29)	21 (35.59)
Unstable job	79 (63.71)	38 (64.41)
Mainly responsible for the economy		
Mother	20 (16.13)	11 (18.64)
Father	70 (56.45)	20 (33.89)
Parental	20 (16.13)	12 (21.05)
Grandparents	14 (11.29)	3 (5.08)
Other members	0 (0)	11 (18.64)
Social assistance	0 (0)	2 (3.39)
Pre-diagnosis costs, Ten thousand yuan	7000 (3000, 15,000)	6000 (4000, 10,000)
Average cost of treatment per month (RMB)	3000 (2000, 5100)	3100 (2200, 5200)

## Discussion

This study provides a comprehensive overview of mitochondrial diseases in China, emphasizing the demographic characteristics, diagnostic challenges, treatment patterns, and caregiver burden. The findings underscore the complexity of mitochondrial diseases and highlight the need for improved diagnostic accuracy, targeted treatment strategies, and comprehensive support systems for patients and their families.

The study revealed a high incidence of mitochondrial disease, as well as difficulties in diagnosis and significant delays. This study reveals a notably high incidence, substantial delays in obtaining confirmative diagnoses, and the necessity for multiple hospital visits before reaching a conclusive diagnosis of mitochondrial disease. A recent case report [22] stated that two cases of MT-ND5-related mitochondrial disease were misdiagnosed as seronegative neuromyelitis optical spectrum disorder. Additionally, misdiagnosis occurred in cases of myasthenia gravis and mitochondrial myopathies due to the presence of shared symptoms [23]. These findings underscore the critical importance of enhancing both basic and clinical knowledge of mitochondrial diseases in China. This will not only contribute to early and accurate diagnoses but also facilitate the implementation of appropriate treatments.

Individuals with mitochondrial disease experience diverse levels of motor, sensory, communicative, and intellectual challenges, significantly impacting their daily life quality [16, 24–26]. A substantial number of mitochondrial disease patients face difficulties in self-care due to limitations in both motor and cognitive functions. This study revealed that over half of the patients with mRS scores ranging from 3 to 5 require assistance from others to accomplish fundamental tasks. Our center’s previous research has conducted relevant studies on the survival analysis of MELAS patients, and the results show that these patients also have a higher mortality rate [27]. Caring for a child with mitochondrial disease places a considerable burden on all family members, with the primary caregiver, especially the mother [28]. This phenomenon may be attributed to maternal instincts and emotional bonds with their children.

Mitochondrial disease also significantly causes the psychological stress in both patients and caregivers [29–31]. In the present study, almost half of the family members expressed a need for professional psychological counseling to address issues such as depression, anxiety, and other mental health concerns. In pediatric mitochondrial disease cases, mothers of patients reported poorer physical and psychological health compared to mothers of children with epilepsy [29]. The elevated occurrence of depression and/or anxiety among mothers of pediatric patients could be partly linked to genetic factors [32]. Regardless of the causative factors, further research is essential to determine whether supportive services can effectively reduce anxiety and stress levels among mothers or other family members of pediatric patients.

Moreover, the lack of information on mitochondrial disease has added to the burden of caregivers and negatively impacted their quality of life [21, 29]. This survey revealed that patients or their families require a more comprehensive understanding of disease-related information, such as effective strategies for treatment and disease management.

Given the clinical diversity and unpredictable course of mitochondrial disease, creating a standardized guideline for treatment and management seems to be unfeasible. Instead, individualized plans should be implemented to address the unique needs of patients and provide education for both patients and caregivers [28]. The collaboration of various healthcare professionals - including metabolic physicians, neurologists, cardiologists, endocrinologists, gastroenterologists, nephrologists, intensivists, ophthalmologists, audiological physicians, community pediatricians, dietitians, nurses, physiotherapists, speech and language therapists, and psychologists - is essential to optimize clinical management and effectively navigate potential complications [33].

In this study, it was found that 41.35% of pediatric patients and 39.39% of adult patients’ families derived their income from unstable jobs. Mitochondrial disease imposes a substantial economic burden on both patients and their families, encompassing various costs related to diagnosis, treatment, and long-term care. The progressive and unpredictable nature of mitochondrial disease challenges the provision of long-term care [28]. The gold standard genetic testing for diagnosis is costly and often not covered by health insurance [16]. Given the recurrent nature of the illness and the need for extended treatment, family members bear an economic burden. In a previous report, 42% of family members underwent significant job changes due to their responsibilities in caring for a medically fragile patient at home [34]. Even with good health insurance, families remained vulnerable to life-altering changes [35–37]. 50.85% of the adult patients in our study had a high school or college education, however, further investigation is needed on the employment situation of patients after graduation.

To date, there is no effective treatment for mitochondrial disease. Supplementary therapies, such as antioxidants and cofactors, are employed [38]. In recent years, various complementary and alternative therapies, encompassing health practices and non-conventional products, have been explored [39]. In the current study, the predominant over-the-counter remedies were dietary supplements, succeeded by antiepileptic drugs and various vitamins. There is a lack of studies supporting the efficacy of high-dose multivitamins, except for coenzyme Q10 [40].

The average yearly cost of complementary and alternative medicine (CAM) in the Netherlands was 489 EUR for pediatric patients and 359 EUR for adult patients [39], which were lower than those in this study. In another prior study, the associated direct medical costs amounted to 113 million USD for approximately 3200 pediatric hospitalizations (1.9 per 100,000 population) and 2000 adult hospitalizations (0.8 per 100,000 population) for mitochondrial disease (mitochondrial disease) in the United States in 2012 [41]. Estimating the cost is challenging due to the disease’s heterogeneity, encompassing different ages of onset, varying symptom severity, and diverse prognoses.

Developing new therapies for mitochondrial disease may reduce hospitalizations and surgeries [16]. The management of mitochondrial disease should encompass not only efficient medications but also environmental enrichment, enhanced rehabilitation, and improved social conditions for disabled patients. Additionally, it should involve providing financial, educational, and psychological support for their families or caregivers [29, 42].

However, this study had several limitations. Firstly, it was a single-center study with a limited sample size of questionnaire survey responses. The symptoms’ manifestation, information needs, economic and psychological impacts reported by the study participants may not be readily extrapolated to the broader population with mitochondrial disease. Secondly, being a questionnaire-based study, respondent bias could influence the outcomes, potentially resulting in either overestimation or underestimation of perceptions regarding treatment effects and the disease course. Lastly, data on incidence, prevalence, and disability-adjusted life years were not calculated in this study.

## Conclusions

In conclusion, this study provides a comprehensive overview of the demographic and disease burden of individuals with mitochondrial disease, which may contribute valuable insights into the challenges faced by individuals and families affected by mitochondrial disease, emphasizing the necessity for holistic and tailored approaches in both clinical management and support services.

## Data Availability

All data generated or analysed during this study are included in this published article and its supplementary information files.

## Abbreviations

MELAS: stroke-like episodes
KSS syndrome: Kearns-Sayre syndrome
CPEO: chronic progressive extraocular muscle paralysis
CAM: complementary and alternative medicine
